# Data on the highly diverse plasma response to a drink containing nutrients

**DOI:** 10.1016/j.dib.2020.105309

**Published:** 2020-02-20

**Authors:** Sandra Unterberger, Alexandra Maier-Salamon, Walter Jäger, Barbara Wessner, Karl-Heinz Wagner

**Affiliations:** a Research Platform Active Ageing, University of Vienna, Austria; b Department of Clinical Pharmacy and Diagnostics, University of Vienna, Austria; c Centre for Sport Science and University Sports, University of Vienna, Austria; d Department of Nutritional Sciences, University of Vienna, Austria

**Keywords:** Bioavailability, Folate, Vitamin B12, Resveratrol metabolites, Plasma response

## Abstract

Bioavailability of nutrients is highly diverse and depends on a variety of endogenous and exogenous factors in humans. This data article reports on the plasma response of 10 human subjects (5 females, 5 males) to a single dose of a multivitamin drink within 6h (blood taken after 1, 2, 4, and 6h). Nutrients, which were considered in the assessment, were folate (Radioimmuno Assay), vitamin B12 (Radioimmuno Assay) and resveratrol and its plasma metabolites resveratrol-3-*O*-glucuronide (R3G), resveratrol-4′-*O*-glucuronide (R4G), resveratrol-3-*O*-sulfate (R3S) and resveratrol-3-*O*-4′-*O*-disulfate (RD, all HPLC). Biological outcome measures were malondialdehyde (MDA, HPLC) and Ferric Reducing ability potential (FRAP, Microplate reader).

Mean plasma concentration increased over time significantly for folate (p < 0.05, maximum concentration (Tmax) after 2h), R3G, R4G, R3S (all p < 0.05, Tmax after 1h), RD (p < 0.05, Tmax after 2h) as well as MDA, which decreased (p < 0.05, Tmax after 2h). No significant change was observed for vitamin B12 and FRAP. Within this mean development, individual changes of participants were highly diverse such as for folate from +42 to +422%, for MDA from −49 to +30% or vitamin B12 from −4 to +33%. For R4G 4 out of 10 subjects showed even no increase in plasma at all. For R4G plasma response ranged from 0 to 36 ng/ml, for R3G from 0 to 53 ng/ml or for R4S from 62 to 265 ng/ml. There was no gender difference regarding the plasma response.

**Table:** Specifications Table

Subject	Nutrition
Specific subject area	Bioavailability of nutrients after multivitamin drink intake
Type of data	Text file, figure
How data were acquired	Radioimmuno Assay for B12 and Folate (Berthold LB 2111 gamma counter) Absorbance for FRAP (BMG FLUOstar OPTIMA Microplate Reader (BMG LABTECH GmbH) HPLC for MDA (LaChrom Merck Hitachi Chromatography System) HPLC for Resveratrol (Dionex UltiMate 3000 HPLC System)
Data format	Raw and graph
Parameters for data collection	Human intervention, plasma response to a single dose
Description of data collection	A concentrated multivitamin beverage (33 ml equivalent to a recommended daily dose) was given to 10 subjects and blood was taken as baseline after 1, 2, 4, and 6h. Folate, vitamin B12 and resveratrol and its plasma metabolites resveratrol-3-*O*-glucuronide (R3G), resveratrol-4′-*O*-glucuronide (R4G), resveratrol-3-*O*-sulfate (R3S) and resveratrol-3-*O*-4′-*O*-disulfate (RD) were assessed at all time points. Biological outcome measures were MDA and FRAP. The protocol was approved by the ethics committee of the University of Vienna.
Data source location	Institution: Department of Nutritional Sciences, University of Vienna City/Town/Region: Vienna, 1030 Country: Austria
Data accessibility	All raw data are given along with the article as supplementary file.

**Table:** 

**Value of the Data** • The data of this article are useful for all readers interesting in the plasma response to a single dose or bioavailability of nutrients and related biological outcomes. • Plasma responses based on a single dose administration and the specific focus on the large plasma variation is interesting for researcher in health care, health education, clinical medicine but also food sciences. • Biological effects after a single dose administration or a short intervention of a supplement/food are sometimes not significant, which is often due to diverse individual plasma responses of the active compounds, responsible for biological effects such as vitamins or active plant compounds. Therefore, it is important to focus on the concept of hypo- and hyper-responder in every related study, in order to better understand the potential of a supplement/food for improved biological functions or health effects.

## 1 Data description

The present data focuses on the average and individual plasma bioavailability of human adult males and females after the intake of a single dose of a multivitamin drink. Bioavailability of small and medium molecules can vary widely in humans [[1], [2], [3]], which in further consequence lead to heterogeneous biological biomarker outcome measures [4].

A single dose of a multivitamin drink was consumed from healthy male and female adults and defined biomarker, which were part of the drink, measured in the blood of the participants at baseline and periodically up to 6h after intake.

**Fig. 1:**
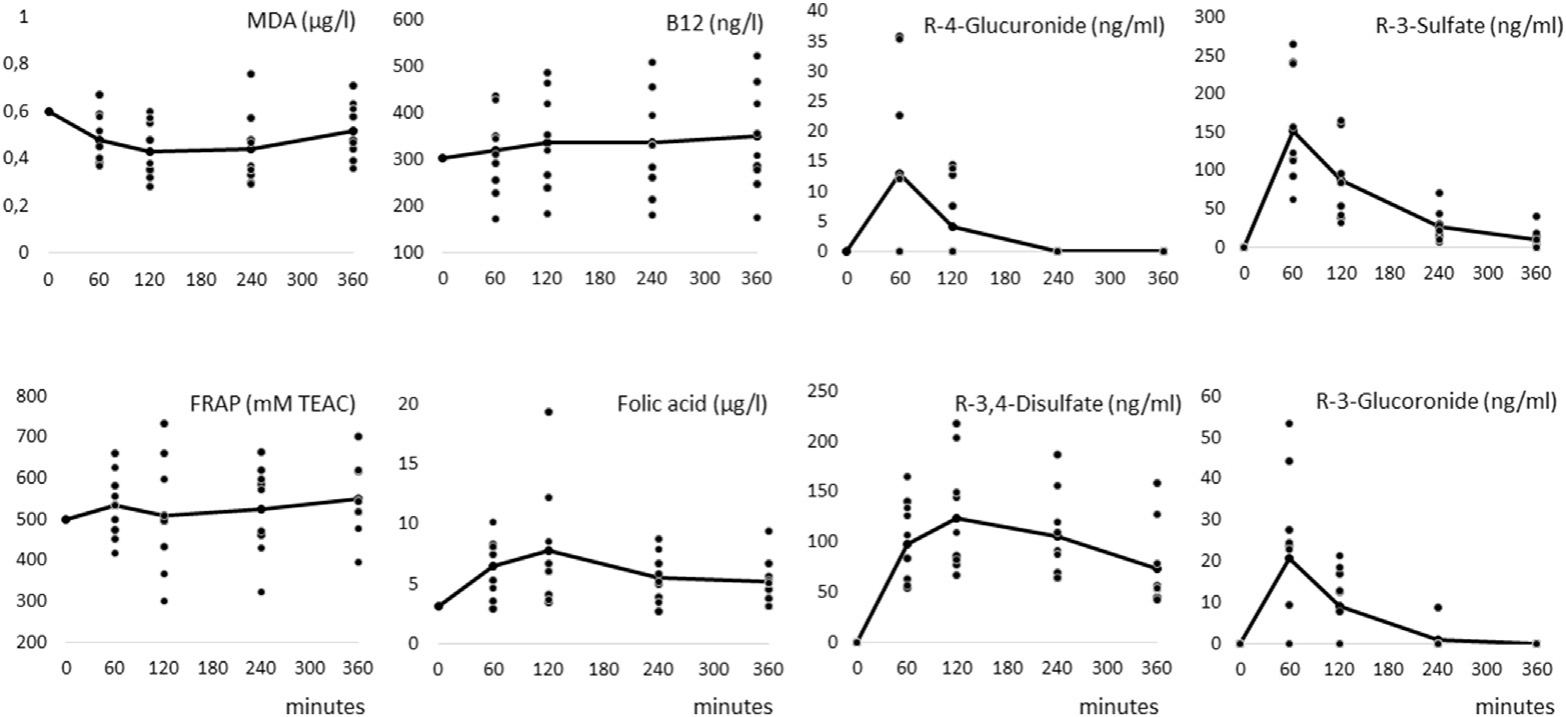
Plasma concentrations of MDA, FRAP, vitamin B12, folic acid, resveratrol-3-*O*-glucuronide (R3G), resveratrol-4′-*O*-glucuronide (R4G), resveratrol-3-*O*-sulfate (R3S) and resveratrol-3-*O*-4′-*O*-disulfate (RD) at baseline and 60, 120, 240 and 360 min after intake of a multivitamin drink. Each point represents the single response of a participant. The full line represents the mean of the group.

Plasma response of folate, vitamin B12 the resveratrol metabolites resveratrol-3-*O*-glucuronide (R3G), resveratrol-4′-*O*-glucuronide (R4G), resveratrol-3-*O*-sulfate (R3S) and resveratrol-3-*O*-4′-*O*-disulfate (RD) as well as the biological outcome parameter MDA and FRAP were measured at every blood sampling time point (Fig. 1).

## 2 Experimental design, materials, and methods

### 2.1 Design and blood sampling

Presented data are coming from a single administration dose response after the intake of 33 ml (equivalent to a recommended daily dose) of a multivitamin beverage (vabo-n essentials ©), which consisted of micronutrients and active plant compounds, among them folate, vitamin B12 and resveratrol. 10 healthy adult volunteers (5 males, 5 females, mean age 29.6 ± 7.5 (21–45) years, mean weight 77,1 ± 22,0 (55–121) kg, mean height 174 ± 12 (158–194) cm), were recruited according to defined inclusion and exclusion criteria. In the morning the drink was administered to the subjects (after a 12h overnight fast) and blood was taken at baseline and after 1, 2, 4 and 6 hours with repeated venepunctures. The baseline plasma levels were for MDA 0,60 ± 0,13 μg/l, for folic acid 3,12 ± 1,3 μg/l, for vitamin B12 303 ± 104 ng/l and for FRAP 500 ± 111 mM TEAC. Resveratrol metabolites were not detectable at baseline.

Written informed consent was obtained from all participants in accordance with the Declaration of Helsinki. The protocol was approved by the ethics committee of the University of Vienna (No. 00204).

Plasma/Serum was separated after centrifugation (10 min, 3.000 rpm) immediately after every blood sampling and stored at −80 °C until analysis.

### 2.2 Inclusion and exclusion criteria

Inclusion: males & females aged: 20–70 years, healthy and without chronic diseases, capable of signing the informed consent, no intake of vitamin and nutritional supplements 4 weeks prior study entry and over the whole study period; constant nutritional and physical activity habits 4 weeks prior study entry and over the whole study period.

Exclusion: males & females younger than 20 years and older than 71 years of age, acute diseases, chronic diseases (cardiovascular, pulmonary and metabolic diseases), pregnant and lactating women, morbid obesity (BMI>40), participation in an other study 30 days prior to study entry, people with organ transplants, subjects with gastrointestinal malabsorption, HIV positive, substance abuse (e.g. alcohol >80 g/d) within the last 2 years, intake of vitamin and nutritional supplements 4 weeks prior study entry and over the whole study period.

### 2.3 Analysis of malondialdehyde (MDA)

MDA levels were determined in duplicates in plasma as described earlier [5]. After heating (60min, 100 °C) plasma samples were neutralized with methanol/NaOH, centrifuged (3min, 3000 rpm) and MDA was measured with high-performance liquid chromatography (HPLC) (excitation: λ = 532nm, emission: λ = 563nm, mobile phase: phosphate buffer:methanol = 60:40, HPLC column 125 × 4 mm, 5 μm; Merck, Vienna, Austria).

### 2.4 Analysis of the ferric reducing ability potential (FRAP)

The antioxidant capacity of serum was measured by performing the ferric reducing ability potential (FRAP) assay as described by Benzie and Strain [6] in triplicates using trolox as standard. Absorbance was measured with at 593 nm and data are expressed as trolox equivalents in mM TEAC.

### 2.5 Analysis of folic acid and vitamin B12

Plasma concentrations of vitamin B12 and folate in plasma were measured by using radioimmunoassay. Standard curves were drawn and sample values calculated according to the protocol published by the kit producer (MP Biomedicals, Germany).

### 2.6 Analysis of resveratrol metabolites

Resveratrol and its glucuronidated and sulfated biotransformation products were quantified by HPLC as described previously [7] using a Dionex UltiMate 3000 HPLC system equipped with an L-7250 injector, an L-7100 pump, an L-7300 column oven (set at 15 °C), a D-7000 interface and an L-7400 UV detector (Thermo Fisher Scientific, Waltham, Massachusetts) set at a wavelength of 307 nm. Separation was done on a Hypersil BDS-C18 column (5 μm, 250 × 4.6 mm I.D., Thermo Fisher Scientific, Inc., Waltham, MA), preceded by a Hypersil BDS-C18 guard column (5 μm, 10 × 4.6 mm I.D.) at a column temperature of 15 °C using a mobile phase consisting of a continuous gradient mixed from 5 mM ammonium acetate/acetic acid buffer, pH 7.4, and methanol at a flow rate of 1 ml/min. Calibration of the chromatogram was accomplished using the external standard method. Linear calibration curves were performed by spiking drug-free cell culture medium with standard solutions of resveratrol, resveratrol-3-*O*-sulfate, resveratrol-3-*O*-4′-*O*-disulfate, resveratrol-3-*O*-glucuronide and resveratrol-4′-*O*-glucuronide to give a concentration range from 0.001 to 10 μg/ml (average correlation coefficients: >0.999). For this method the lower limit of quantification for resveratrol and resveratrol conjugates was 5 ng and 7 ng, respectively. Coefficients of accuracy and precision for these compounds were <11%.

The outcome of all parameters are shown in Fig. 1. Raw data are shared as supplementary file.

### 2.7 Statistical analysis

Statistical analyses were performed using IBM SPSS Statistics 23. To assess the overall differences between the time points, the Friedman test was conducted and if significant, the Wilcoxon test was used to calculate the differences between each time point by considering Bonferroni correction. The data are expressed as mean ± SEM (MIN – MAX). P < 0.05 was considered as statistically significant.

## Funding

This work was supported by the University of Vienna and Vabo-N. None of the funders has a financial interest.
